# Gastric Metastasis From Clear Cell Renal Cell Carcinoma: A Case Report

**DOI:** 10.7759/cureus.82801

**Published:** 2025-04-22

**Authors:** Nuno Gonçalves, Cristina Monteiro, Luísa Calais Pereira, João Pedro Mendes, José Paulo Couto

**Affiliations:** 1 General Surgery, Unidade Local de Saúde do Alto Minho, Viana do Castelo, PRT

**Keywords:** case report, gastric metastasis, gastrointestinal bleeding, palliative care, radiotherapy, renal cell carcinoma

## Abstract

Gastric metastases from solid tumors are rare and often clinically silent. When present, they typically originate from primaries such as the lung, breast, or skin. Renal cell carcinoma (RCC), particularly the clear cell subtype, has a known metastatic potential, most commonly to the lungs, bones, and liver. Gastric involvement is extremely uncommon.

We report a case of an 83-year-old man with a remote history of clear cell RCC treated with nephrectomy three decades earlier, who presented with melena, epigastric discomfort, and vomiting. Endoscopic evaluation revealed a conglomerate of friable, vegetative lesions in the stomach with signs of recent bleeding. Biopsies confirmed metastasis of clear cell RCC. Computed tomography (CT) imaging demonstrated multiple nodular intraluminal gastric lesions. Given the patient’s advanced age, comorbidities, and known distant metastases, surgical treatment was deemed inappropriate. Palliative hemostatic radiotherapy was initiated for symptom control.

This case highlights the potential for late gastric metastasis from clear cell RCC and emphasizes the importance of considering metastatic disease in the differential diagnosis of gastrointestinal bleeding, even decades after the initial oncologic treatment. Prompt endoscopic evaluation and histological confirmation are essential for diagnosis, while treatment decisions must be individualized based on disease burden and patient condition.

## Introduction

Gastric metastases from solid tumors are rare, with an estimated incidence ranging from 0.2% to 0.7% [1]. They are usually hematogenous in origin and often arise from primary tumors of the lung, breast, esophagus, skin (melanoma), cervix, testicles, or colon [2]. Renal cell carcinoma (RCC), particularly the clear cell subtype, typically metastasizes to the lungs, bones, and liver [3]. Gastric involvement is extremely uncommon and often underdiagnosed, as symptoms may be nonspecific or absent [4]. In such cases, upper gastrointestinal bleeding or anemia may be the first indication of metastatic spread. With increased long-term survival following curative nephrectomy, unusual metastatic patterns may emerge even years after initial treatment. We present a rare case of gastric metastasis from clear cell RCC, presenting with upper gastrointestinal bleeding and constitutional symptoms, decades after curative nephrectomy.

## Case presentation

An 83-year-old male presented to the emergency department with a three-week history of melena, epigastric discomfort, early satiety, and non-bilious, non-bloody vomiting. He reported no weight loss or dysphagia. His past medical history was significant for clear cell RCC, for which he underwent a left nephrectomy in 1994. He had known hepatic and contralateral renal metastases that had been mostly stable under sunitinib for over nine years. Other comorbidities included arterial hypertension, dyslipidemia, benign prostatic hyperplasia, hypothyroidism secondary to sunitinib, osteoarticular disease, and a history of inguinal hernioplasty.

On physical examination, the patient was pale but hemodynamically stable: blood pressure 125/80 mmHg, heart rate 80 bpm, temperature 36°C, and oxygen saturation 98% on room air. Abdominal examination was unremarkable.

**Figure 1:**
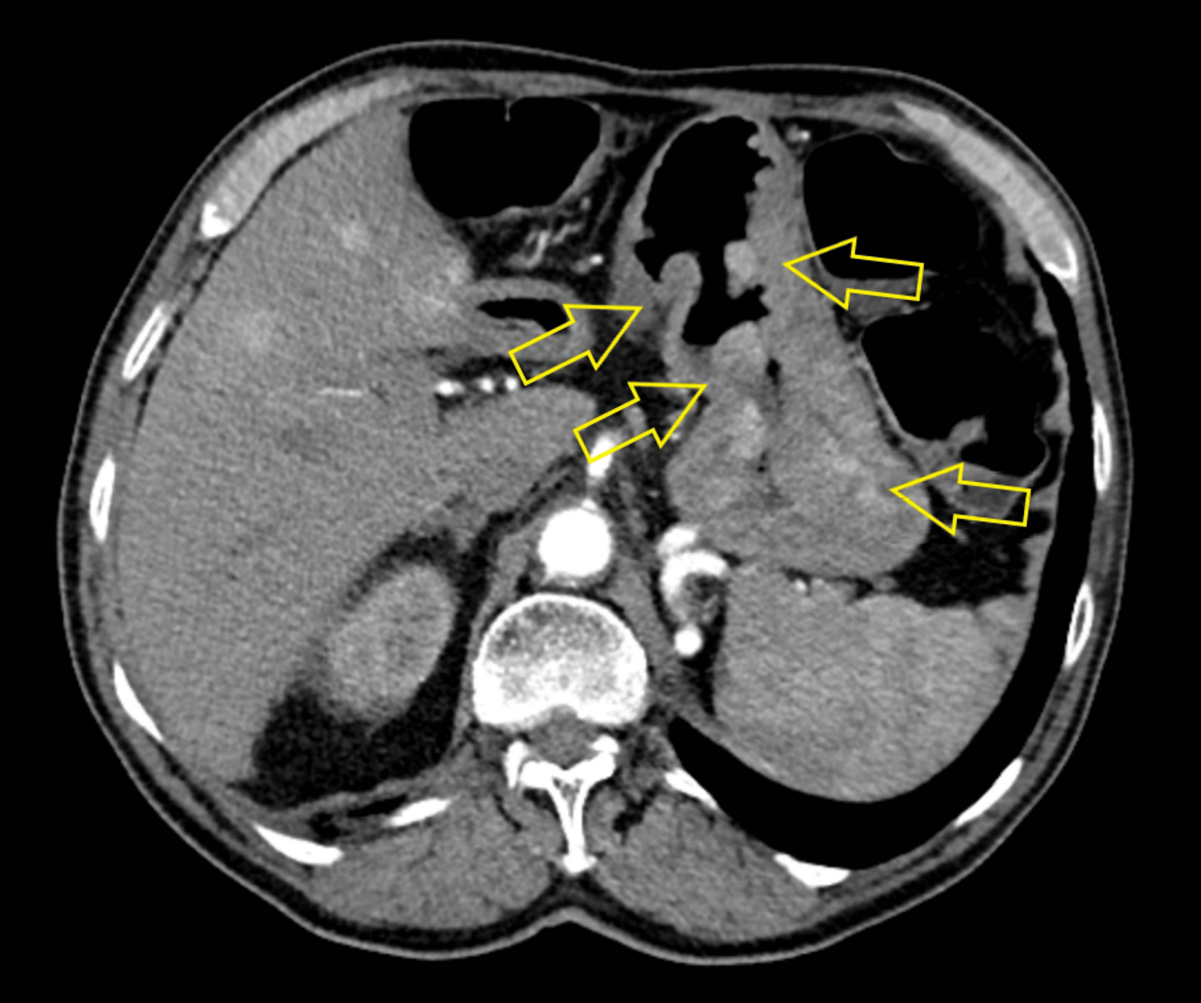
Axial contrast-enhanced CT image showing multiple nodular lesions in the gastric wall (yellow arrows).

Laboratory tests revealed normocytic normochromic anemia with a hemoglobin level of 6.3 g/dL (last measured less than one month earlier with a hemoglobin level of 9.7 g/dL). He received red blood cell transfusions and supportive therapy. A contrast-enhanced computed tomography (CT) scan of the chest, abdomen, and pelvis revealed multiple nodular intraluminal gastric lesions up to 40 mm in size, without signs of gastric wall perforation or obstruction (Figure 1).

**Figure 2:**
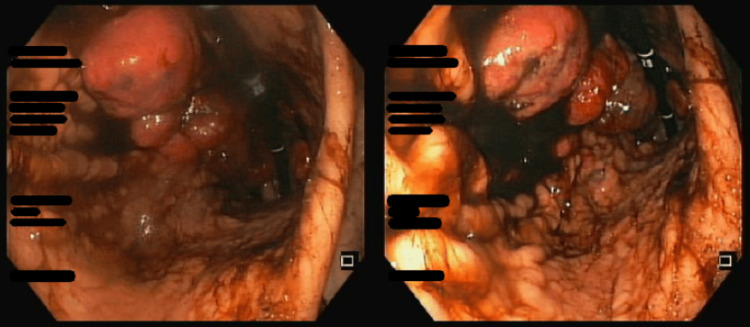
Esophagogastroduodenoscopy showing friable, vegetative lesions extending from the gastric cardia to the body.

**Figure 3:**
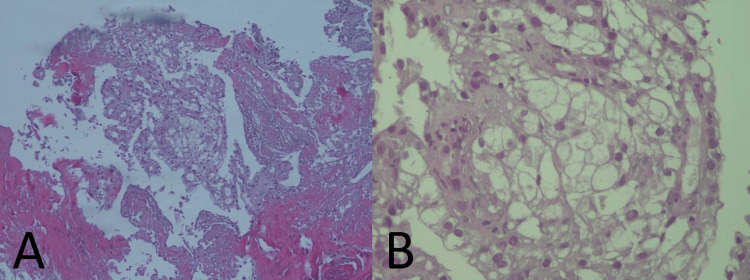
Histopathological examination of gastric biopsy. A: (10X) hematoxylin-eosin staining showing proliferation of clear cells with acinar architecture; B: (40X) proliferation of clear cells with small nucleus and occasional nucleolus.

Esophagogastroduodenoscopy (EGD) revealed a large conglomerate of friable, vegetative lesions with signs of recent bleeding, extending from the cardia throughout the body of the stomach (Figure 2). The lesions were not amenable to endoscopic resection. Biopsies were obtained. Histopathological examination showed malignant epithelial proliferation with solid and acinar patterns composed of clear cells, consistent with gastric metastasis of clear cell RCC (Figure 3).

Given the patient's age, comorbidities, and extent of metastatic disease, surgical resection was not proposed. Palliative hemostatic radiotherapy was administered for symptom control. After stabilization and tolerance to oral intake, the patient was discharged with outpatient follow-up. Over the course of one year of follow-up, the patient has remained clinically stable, requiring occasional red blood cell transfusions due to persistent but manageable anemia.

## Discussion

Although RCC commonly metastasizes to the lungs, liver, and bones, gastric metastases are exceedingly rare. In a systematic review by Eslick and Kalantar, fewer than 50 cases of gastric metastasis from RCC had been documented in the literature at the time of publication [4]. Most patients present with gastrointestinal bleeding, anemia, or nonspecific abdominal symptoms, although some may be asymptomatic.

The latency between nephrectomy and gastric metastasis can be remarkably long, often exceeding a decade, suggesting the ability of RCC to remain dormant before reactivating in distant sites [5]. This case is notable for its exceptionally delayed presentation, occurring over 30 years after the initial nephrectomy.

Diagnosis is typically established via endoscopic biopsy. The presence of clear cells with solid and acinar architecture strongly supports the diagnosis of metastatic clear cell RCC (ccRCC). Imaging studies, particularly contrast-enhanced CT, can further delineate the extent of the disease and identify additional metastatic sites.

There is no standardized treatment for gastric metastases of RCC due to the rarity of the condition. Therapeutic approaches depend on the presence of systemic disease, patient performance status, and severity of symptoms. In patients with isolated gastric metastasis and good performance status, surgical resection may be considered and has been associated with prolonged survival in selected cases [6]. However, in disseminated disease or in frail patients, palliative measures such as radiotherapy or embolization may be more appropriate for symptom control, especially in cases of gastrointestinal bleeding.

In our case, the decision to avoid surgery was based on the patient’s advanced age, comorbidities, and known disseminated metastases. Hemostatic radiotherapy was chosen as a means of palliation, with the goal of minimizing further bleeding and improving quality of life.

This case highlights the importance of considering late gastric metastasis in patients with a remote history of RCC who present with upper gastrointestinal symptoms. It also emphasizes the need for individualized, multidisciplinary management strategies in such rare clinical scenarios.

## Conclusions

Gastric metastasis from clear cell RCC is exceptionally rare, often presenting many years after the initial diagnosis. This case illustrates the importance of considering metastatic disease in patients with a remote history of RCC and new-onset gastrointestinal symptoms. Early recognition and appropriate palliative management can help control symptoms and improve patient quality of life, especially in advanced disease settings.
